# Lipoic Acid Conjugated Boron Hybrids Enhance Wound Healing and Antimicrobial Processes

**DOI:** 10.3390/pharmaceutics15010149

**Published:** 2022-12-31

**Authors:** Hasan Türkez, Özge Çağlar Yıldırım, Sena Öner, Abdurrahim Kadı, Abdulkadir Mete, Mehmet Enes Arslan, İrfan Oğuz Şahin, Ömer Erkan Yapça, Adil Mardinoğlu

**Affiliations:** 1 Department of Medical Biology, Faculty of Medicine, Atatürk University, 25240 Erzurum, Turkey; 2 Department of Molecular Biology and Genetics, Erzurum Technical University, 25050 Erzurum, Turkey; 3 Department of Pediatrics, Pediatric Cardiology, Faculty of Medicine, Ondokuz Mayıs University, 55139 Samsun, Turkey; 4 Department of Gynecology and Obstetrics, Faculty of Medicine, Atatürk University, 25240 Erzurum, Turkey; 5 Science for Life Laboratory, KTH-Royal Institute of Technology, SE-17121 Stockholm, Sweden; 6 Centre for Host-Microbiome Interactions, Faculty of Dentistry, Oral & Craniofacial Sciences, King’s College London, London SE1 9RT, UK

**Keywords:** wound healing, antimicrobial effect, nanoparticles, lipoic acid, boron nitride, boron carbide

## Abstract

Complications of chronic non-healing wounds led to the emergence of nanotechnology-based therapies to enhance healing, facilitate tissue repair, and prevent wound-related complications like infections. Here, we design alpha lipoic acid (ALA) conjugated hexagonal boron nitride (hBN) and boron carbide (B_4_C) nanoparticles (NPs) to enhance wound healing in human dermal fibroblast (HDFa) cell culture and characterize its antimicrobial properties against *Staphylococcus aureus* (*S. aureus*, gram positive) and *Escherichia coli* (*E. coli*, gram negative) bacterial strains. ALA molecules are integrated onto hBN and C_4_B NPs through esterification procedure, and molecular characterizations are performed by using transmission electron microscopy (TEM), Fourier transform infrared spectroscopy (FTIR), and UV-vis spectroscopy. Wound healing and antimicrobial properties are investigated via the use of cell viability assays, scratch test, oxidative stress, and antimicrobial activity assays. Based on our analysis, we observe that ALA-conjugated hBN NPs have the highest wound-healing feature and antimicrobial activity compared to ALA-B_4_C. On the other hand, hBN, ALA-B_4_C, and ALA compounds showed promising regenerative and antimicrobial properties. Also, we find that ALA conjugation enhances wound healing and antimicrobial potency of hBN and B_4_C NPs. We conclude that the ALA-hBN conjugate is a potential candidate to stimulate regeneration process for injuries.

## 1. Introduction

Wounds disrupt the integrity of the skin or mucosa by various internal or external factors [1]. Wound healing is a complex and dynamic processes in the human body; it replaces devitalized and missing cellular structures and tissue layers. The long process of wound healing starts from the formation of the wound and can take months or years. This process can be positive or negative [2]. The critical factor in the healing is to provide perfusion and oxygen exchange and to feed the scar tissue a sufficient amount [3]. Local or systemic factors such as age, nutrition, and smoking may also affect the wound-healing process. Infections are common during wound healing. When the wound is formed, it is very easy for microorganisms to reach the lower tissues. If the scar tissue is not cleansed of microorganisms, more cytokines are released and the recovery of simple injuries may take longer and turn into chronic wounds [4].

Non-healing wounds and skin regeneration represent a huge socio-economic burden and there is an immediate need for innovative approaches for successful wound management. The recent developments in nanotechnology enabled scientists to provide solutions on wound healing and skin regeneration [5]. It has been reported that the use of nanosystems like growth factor-loaded polymeric networks may accelerate the wound-healing process due to their unique compatibility of nanosystems with the skin and the moisture provided [6]. Also, silver (Ag)-based nanosystems were shown to enhance the wound-healing process and inflectional status in diabetes-related persistent wounds [7].

Numerous studies have been performed on the development and testing of different compounds to enhance the healing process. Although each molecule accelerates the wound healing machinery, it was observed that all of these processes comes with a problem to be solved [8]. It has been reported that boron compounds have beneficial properties for healing and have great potency in the wound-healing process. Previous studies reported that boric application improves the deep wound-healing process nearly two-fold compared to other antiseptic applications [9,10]. Furthermore, boron compounds have been proposed for use in wound healing due to their other advantageous properties beside regenerative functions. Pharmacopeia, which is known as boric acid, has been utilized as an antimicrobial agent and showed promising results in healing infections. On the other hand, side effects of pharmacopeia in ingestion limits its use in different medical applications [11,12]. Thus, it is necessary to develop boron compounds that can be used as a regenerative and antimicrobial compound without the undesired side effects in the treatment of wound healing.

Here, we investigated the wound healing and antibacterial activity of hBN and B_4_C nanoparticles conjugated with ALA on the fibroblast cells and bacterial cell cultures. ALA was chosen to integrate the nanoparticle system because ALA have better properties than LA like stability in physiological environments and greater tissue absorption [13]. We first synthesized ALA-conjugated nanoparticles via the esterification reaction and performed molecular characterizations by using TEM, FTIR, and UV-vis spectroscopy methods. Second, we performed MTT cell viability and scratch assays to analyze regenerative properties of synthesized compounds. Next, we used an antimicrobial activity test to investigate infection-preventive features of the nanoparticles on *S. aureus* and *E. coli* bacterial strains. Finally, we explored potential regenerative and antiseptic properties of the boron compound in the wound-healing process.

## 2. Materials and Methods

### 2.1. Materials

Alpha- Lipoic Acid (ALA), EDC (CAS No 25952-53-8), DMAP (CAS No 1122-58-3), and N,N,dimetilasetamide (CAS No 127-19-5) were purchased from Sigma-Aldrich (St. Louis, MO, USA). Dulbecco’s Modified Eagle Medium: Nutrient Mixture F-12 (DMEM/F-12), fetal bovine serum (FBS) and penicillin-streptomycin (5000 U/mL) were purchased from Thermo Fisher Scientific, Waltham, MA, USA. All other chemicals were purchased from Sigma-Aldrich.

### 2.2. Conjugations of Alpha Lipoic Acid (ALA) onto hBN and C_4_B Nanoparticles

A concentration of 0.5 mg/mL of ALA was attached onto the hBN and B_4_C particles by the esterification reaction. The reaction was carried out with minor modifications of our previous work [14]. Briefly, 30 mg of hBN and B_4_C were mixed with 30 mL HNO_3_ for 1 h and stirred overnight. The mixture was washed twice with dH_2_O using centrifugation (Thermo Fisher Scientific, Waltham, MA, USA). The pellet was dried at room temperature. Thirty milligrams of N-(3-dimethyl aminopropyl)-N’-ethyl carbodiimide hydrochloride (EDC) and 20.1 mg 4 (dimethylamino)-pyridine (DMAP) were suspended in 60 mL N, N-dimethylacetamide (99.8%), then the sample was mixed for 30 min with 30 mg ALA. The samples were stirred at room temperature overnight. The hBN-ALA and B_4_C-ALA were collected by centrifugations. Then the samples were washed twice with dH_2_O.

### 2.3. Characterizations of Nanoconjugates

The morphology and particle size of hBN, B_4_C, hBN-ALA, and C_4_B-ALA were investigated using transmission electron microscopy (EOL JEM-ARM200CFEG UHR-TEM, Tokyo, Japan). Fourier transform infrared spectroscopy (Bruker VERTEX 70v. FTIR) was used to validate the interaction of hBN and C_4_B nanoparticles with ALA. Finally, all the samples were characterized by UV-vis spectroscopy (Epoch™, Biotek, Winooski, VT, USA).

### 2.4. Human Dermal Fibroblasts (HDFa) Cell Culture and Cell Viability Analysis

Human dermal fibroblast cells (HDFa, ATCC PCS-201-012, Manassas, VI, USA) were cultured in Dulbecco’s Modified Eagle’s medium (Sigma-Aldrich, DMEM), containing 10% FBS and 1% penicillin-streptomycin. Cells were kept in a cell culture incubator at 37 °C and 5% CO_2_ until reaching 80% confluency. Cells were washed twice with 5 mL PBS. Then, trypsin was added to the Petri dish. Cells were removed by centrifugation at 5000 rpm for 3 min then the pellet was dissolved with fresh DMEM medium.

All cells were counted using a hemocytometer. Cells were seeded into a 48-well plate as 10^5^ cells in each well. The well-plate was kept in a cell culture incubator for 24 h. Cytotoxicity was determined by using an MTT reagent with a colorimetric analysis [15]. For this purpose, hBN, hBN-ALA, B_4_C, B_4_C-ALA, and ALA compound were prepared at specific concentrations (1.6–200 µg/mL) and added to the wells separately in triple replicates. The plate was incubated for 24 h at 37 °C and 5% CO_2_. Then, the medium was discarded and MTT reagent prepared within PBS was added to each well. The culture was placed in an incubator at dark for 3 h. Formazan crystals formed by living cells were dissolved with DMSO. The plate was read at 570 nm using a microplate reader [16].

### 2.5. Total Antioxidant Capacity (TAS) and Total Oxidant Status (TOS) 

Antioxidants reduced total antioxidant capacity (TAS). Total Oxidant Status (TOS) evaluations were performed according to the manufacturer’s (Rel Assay Diagnostics^®^, Gaziantep, Turkey) instructions for all groups on the HDFa cell line. Antioxidants reduce ABST to a colorless form. Absorbance evaluations at 660 nm are proportional to the antioxidant level. In this method, Trolox Equivalent is used as the positive control group. In summary, the culture supernatant was treated with Reagent 1 (Buffer Solution-Acetate Buffe) and the first absorbance was read at 660 nm using a microplate reader. Reagent 2 (Prochromogen Solution-ABTS) was added to the medium and the second absorbance values were recorded. The evaluation was made following the kit procedure of the manufacturer. For TOS measurement, the medium was mixed with Reagent 1 (Buffer Solution-H_2_SO_4_) and the plate was read using a microplate reader at 530 nm. Then, Reagent 2 (Substrate Solution-H_2_SO_4_, Ferrous ion, O-dianisidine) was added into the mixture. Absorbance 2 was recorded and values were calculated for all groups.

### 2.6. Wound Healing Test

In vitro scratch assay was carried out according to a previous report. In summary, 10^5^ cells were seeded into a 6-well plate containing DMEM medium and cultivated overnight. Cells were washed twice with PBS and a 200 µL sterile tip was scratched. After washing the cells with DPBS, the detached cells were removed. The cells were treated with 50 µg/mL of hBN, B_4_C, hBN-ALA, B_4_C-ALA, and ALA for 12 h. Morphological changes and cell migrations were observed using light microscopy at the 12th hour.

### 2.7. Antimicrobial Activity

*S. aureus* and *E. coli* were used to determine the antimicrobial activity of hBN, hBN-ALA, B_4_C, B_4_C-ALA, and ALA. Bacterial strains were grown in Mueller Hinton agar (MHA) medium at 37 °C. Then these samples were suspended in Mueller Hinton broth (MHB) medium at 37 °C at 200 rpm. 0.5 McFarland concentration of these bacterial strains were added into 6-wellplate including MHB medium and were added with 50 µg/mL concentrations of hBN, hBN-ALA, B_4_C, B_4_C-ALA NPs, and ALA compound. After the plate was incubated at 37 °C for 24 h, the samples were washed twice with PBS. Finally, the bacterial strains were collected by using trypsin and an OD measurement was performed at 600 nm.

## 3. Results

### 3.1. Characterizations of Nanoconjugates

**Scheme 1:**
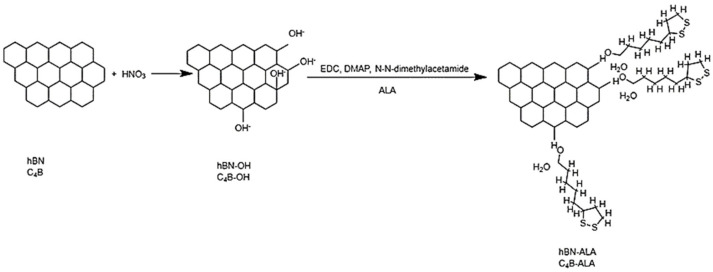
Chemical interaction reaction of LA to hBN and B_4_C.

**Figure 1:**
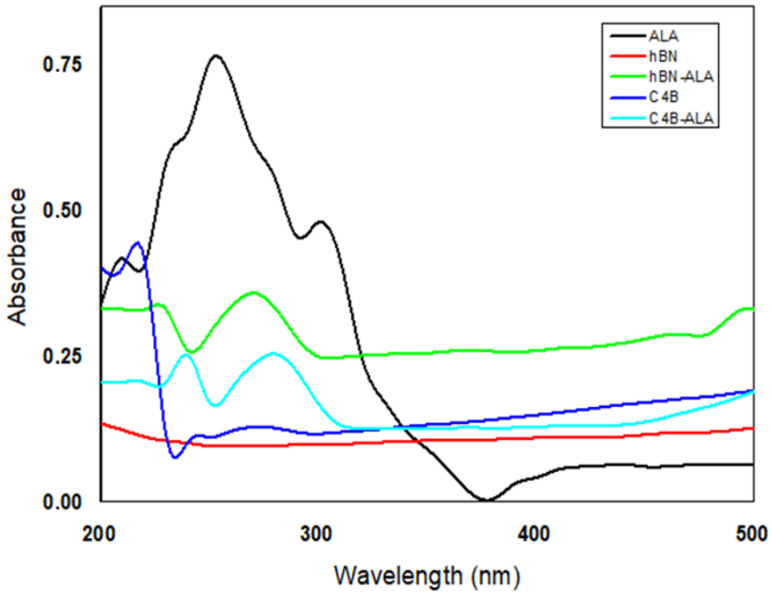
UV-vis spectrum of hBN, B_4_C, hBN-ALA, B_4_C-ALA, and ALA.

**Figure 2:**
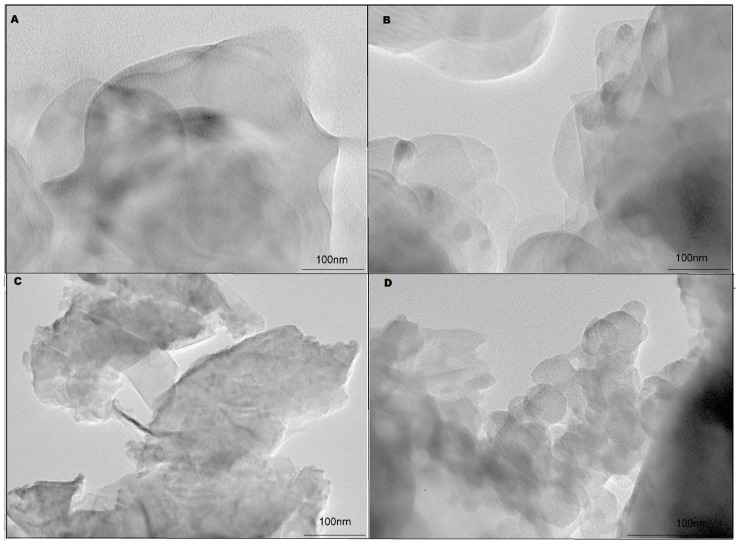
Images (**A**) hBN, (**B**) hBN-ALA (**C**) B_4_C, and (**D**) B_4_C-ALA at 100 nm scale under TEM.

**Figure 3:**
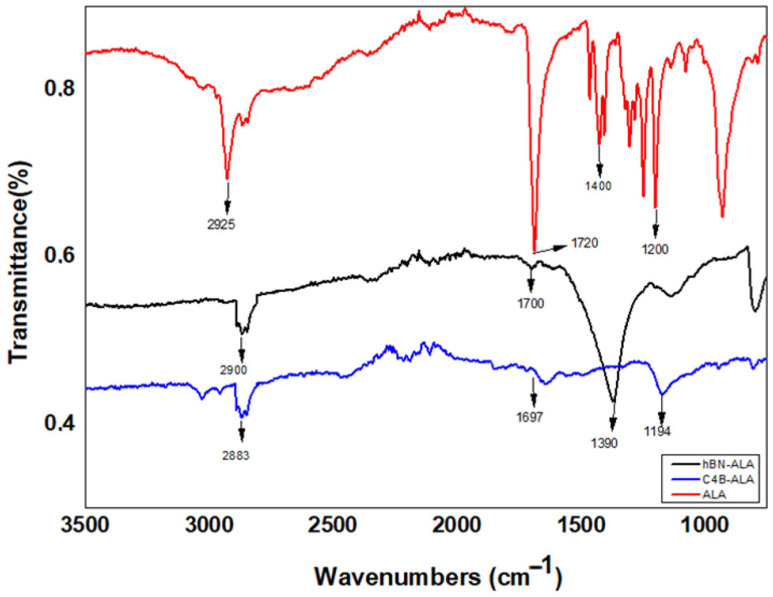
FTIR spectra of hBN-ALA, B_4_C-ALA, and ALA.

Chemical interaction and conjugation of LA to HBN and B_4_C was shown in Scheme 1. In the UV-vis spectrum analysis, the max peak of ALA was taken as the baseline to investigate the conjugation of ALA to the hBN and C_4_B surfaces. Pure ALA exhibited a maximum peak at 253 nm when measured in the 200–800 nm range. hBN and C_4_B samples did not have a peak in this region; the values for hBN-ALA and C_4_B-ALA are 274 nm and 278 nm, respectively (Figure 1). TEM images showed that the hBN and B_4_C nanoparticles had non-uniform morphology and the average particle size was 100–200 nm. There was no significant morphological difference in the ALA group-attached hBN and B_4_C samples, and the average particle size was 200–250 nm (Figure 2). In addition, FTIR analyses were performed to confirm bond variety on hBN and B_4_C nanoparticles with ALA molecules. The results of the FTIR analysis confirming the conjugation of ALA by the esterification reaction to the hBN and C_4_B surface are given in Figure 3. In the sample representing pure ALA, the O-H bond shows a strong vibration at 1200 cm^−1’^ and 1400 cm^−1^, while this stretch band is at 1194 and 1390 cm^−1^ for C_4_B-ALA and hBN-ALA samples, respectively. As a matter of fact, a research report confirmed that the vibrations of tension bands representing ALA are at 1250 cm^−1^ for O-H bond and at 1740 cm^−1^ for C=O [17]. The vibration frequencies of 1697 and 1700 cm^−1^ representing the C=O stretch band belong to the C_4_B-ALA and hBN-ALA samples, respectively. In addition, the symmetrical and asymmetrical bonds of ALA, which were reported as strong vibrations at 2880 and 2920 cm^−1^ in the studies carried out, were in the tension band represented as 2900 cm^−1^ in the hBN-ALA group and 2883 cm^−1^ in the C_4_B-ALA samples [18].

### 3.2. Human Dermal Fibroblasts (HDFa) Cell Culture and Cell Viability Analysis

**Figure 4:**
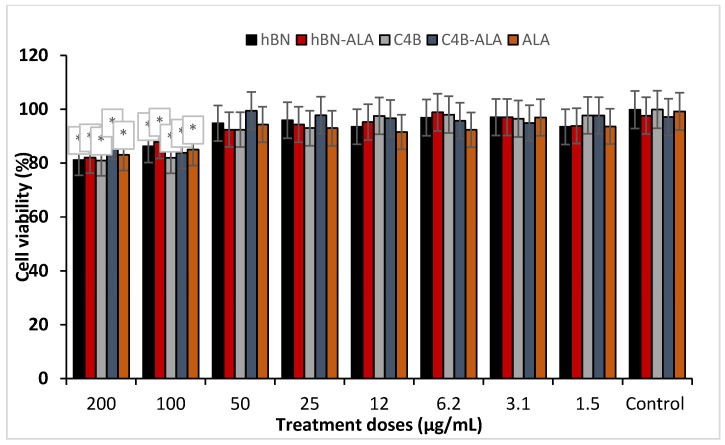
MTT assay in HDF cell cultures for hBN, hBN-ALA, B_4_C, B_4_C-ALA, and ALA at 24 h. Symbol (*) represents statistically significant difference (*p* ≤ 0.05) compared to the negative control.

Cell viability assay was carried out on HDF cell lines to study the effect of concentrations in a wide spectrum range (1.6–200 µg/mL) for hBN, B_4_C, hBN-ALA, B_4_C-ALA, and ALA. It was observed that concentrations of 50 ug/mL and lower concentrations did not cause any significant toxicity on the HDFa cell line. In addition, lower concentrations have an increasing effect on viability compared to the control group. Especially when the B_4_C and B_4_C-ALA groups were compared to the control, we found that ALA attachment significantly reduced the toxicity of B_4_C on the HDF cell line. Hence, we observed that 50 µg/mL concentration is a safe dose for performing the scratch test (Figure 4).

### 3.3. Total Antioxidant Capacity (TAC) and Total Oxidant Status (TOS)

In the TAS and TOS analysis, cell culture supernatants were used to calculate antioxidant and oxidative stress parameters for each experimental group. We found that all of the molecules significantly increased the antioxidant status for the HDFa cell culture compared to the negative control (non-treated). On the other hand, we found that there was no significant change in the oxidative status in cell cultures compared to the negative control (Table 1).

### 3.4. Wound Healing Test

**Figure 5:**
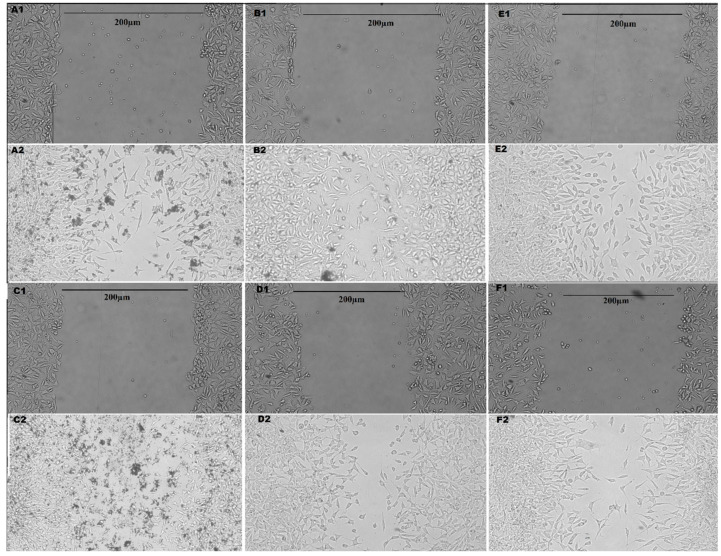
Effects of 50 ug/mL concentrations of hBN, hBN-ALA, C_4_B, C_4_B-ALA, and ALA on HDF cells culture monitored at 20× magnification under the invert microscope. (**A1**) hBN at 0 h, (**A2**) hBN at 24 h, (**B1**) hBN-ALA at 0 h, (**B2**) hBN-ALA at 24 h, (**C1**) B_4_C at 0 h, (**C2**) B_4_C at 24 h, (**D1**) B_4_C-ALA at 0 h, (**D2**) B_4_C-ALA at 24 h, (**E1**) ALA at 0 h, (**E2**) ALA at 24 h, (**F1**) Control at 0 h, (**F2**) Control at 24 h.

**Figure 6:**
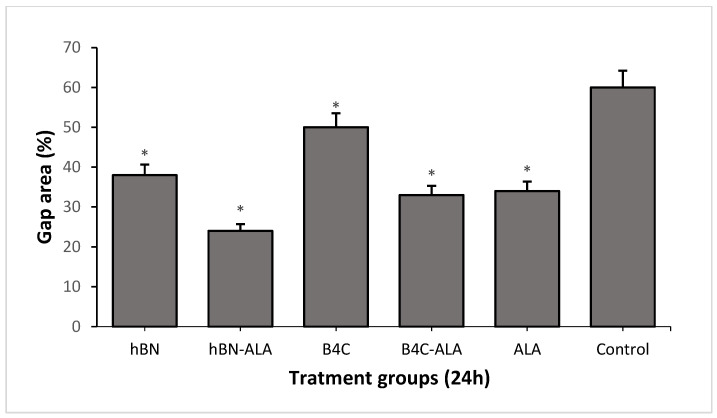
The wound gap distance for the all groups after 24 h. Symbol (*) represents statistically significant difference (*p* ≤ 0.05) compared to the negative control.

Wound-healing effects of hBN, B_4_C, hBN-ALA, B_4_C-ALA, and ALA molecules were investigated on HDFa cell cultures by performing a scratch test. We found that all of the experimental groups showed significantly improved healing properties in wound gap closure when treated with 50 µg/mL of nanoparticles. Also, hBN-ALA nanoparticles showed the highest wound-healing potency compared to other experimental groups. After 24 h of hBN-ALA nanoparticle application, wound gap was closed by nearly 75%, which is a very high ratio compared to the negative control with 40% gap closure. Moreover, we found that ALA conjugation to particles had a significant effect on wound-healing enhancement when compared with non-ALA-conjugated groups (Figure 5 and Figure 6).

### 3.5. Antimicrobial Activity Test

**Figure 7:**
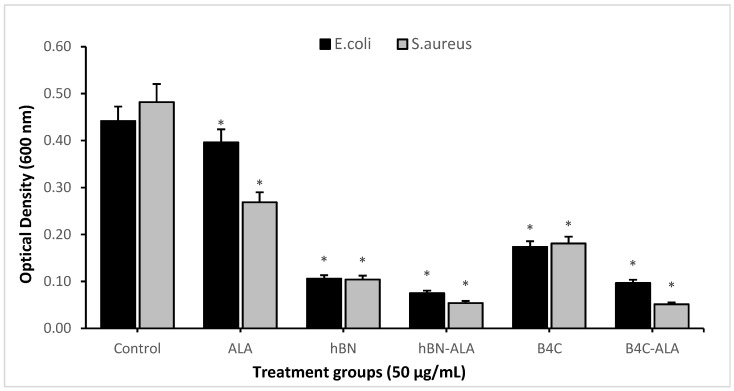
Antimicrobial activity of hBN, hBN-ALA, B_4_C, B_4_C-ALA, and ALA nanoparticles on *S. aureus* and *E. coli* bacterial strains for 24 h. Symbol (*) represents statistically significant difference (*p* ≤ 0.05) compared to the negative control.

Antimicrobial properties of hBN, B_4_C, hBN-ALA, B_4_C-ALA, and ALA nanoparticles (50 µg/mL) were investigated on *E. coli* and *S. aureus* bacterial cultures for 24 h. We found that all of the candidate compounds exhibit strong antimicrobial properties beside the ALA molecule. On the other hand, we observed that ALA conjugation enhances even further antimicrobial properties of B_4_C and hBN nanoparticles against Gram-positive and Gram-negative bacterial strains. Moreover, we observed that hBN-ALA nanoparticles have the highest antimicrobial potential against both of the bacteria species (Figure 7).

## 4. Discussion

The wound-healing process involves different machinery elements like proteins, cytokines, and growth factors. If there are abnormalities in the different steps of the process like disruption in growth factor secretion or gene function related to healing, it leads to extended immunological responses and chronic wound conditions [19]. Although various types of treatments have been suggested to accelerate the healing process such as engineered skin transplantation, growth factor application, hyperbaric oxygen therapy and electrical stimulation, novel chemical formulations are still investigated to aid tissue repair studies since these medical treatments are expensive, require special facilities to apply, are not easily accessible, or have low efficiency [20].

There is an indispensable need for safe and effective treatment options. There is an elevated trend in the use of boron compounds in medicine because of their special properties like high chemical stability, low toxicity, mechanical strength, and absorption of UV light. It was shown in different studies that boron compounds especially boric acid have promising potential in wound-healing therapies, but it has a crucial limitation due to its short half-life [8,21,22,23]. Furthermore, hBN and C_4_B molecules degrade slowly in physiological conditions and modulate the healing process rapidly without causing undesired toxicological outcomes. These properties make hBN and C_4_B compounds promising candidates for wound-healing studies [24].

In this study, an ALA molecule was attached onto hBN and C_4_B nanoparticles through the use of esterification reaction. Integration of ALA into boron compounds was confirmed by using UV-visible spectrum and FTIR analyses. Specific peak arrangement of FTIR analyses showed that ALA groups can be seen at 1371 and 1180 cm^−1^ for hBN-ALA and B_4_C-ALA, respectively. These results were in agreement with the literature data [17].

Based on cytotoxicity analyses, we found that hBN-ALA and B_4_C-ALA nanoparticles exhibit low-level toxicity on the fibroblast cell line and there was no cell viability difference under 50 µg/mL concentrations of hBN-ALA and B_4_C-ALA applications compared to the negative control. Previous studies have also shown that hBN and B_4_C nanoparticles do not elevate cytotoxicity in different types of cell culture under certain treatment concentrations [14,15,25,26,27,28,29]. Besides, antioxidant treatments have been shown to exhibit accelerating features that have been previously used in wound-healing treatment studies [30,31,32,33]. hBN-ALA and B_4_C-ALA conjugates were shown to have antioxidant effects in the HDFa cell cultures and antioxidant features of these compounds promoted the healing process. Also, previous studies confirmed our results that BN and B_4_C molecules exhibit strong antioxidant properties in different physiological conditions [34,35,36,37].

In addition, we investigated the wound-healing properties of hBN, B_4_C, hBN-ALA, B_4_C-ALA, and ALA nanoparticles by using a scratch test on HDFa cell line. We observed that hBN-ALA and B_4_C-ALA conjugates showed superior healing features compared to hBN, B_4_C, and ALA molecules. We also observed that the ALA conjugation synergically enhances regenerative properties of hBN and B_4_C compounds. Previous studies investigated into whether boron-based compounds enhance the wound-healing process through different cellular machinery such as increasing protein, collagen, and proteoglycan synthesis; modulating immune response; and enhancing growth factors [38,39,40,41,42]. Besides, we analyzed effective antimicrobial properties of hBN-ALA and B_4_C-ALA conjugates against *E. coli* and *S. aureus* bacterial cultures. We found that hBN-ALA and B_4_C-ALA nanoparticles are effective antimicrobials against both Gram-negative and Gram-positive bacterial strains. It was also shown in previous studies that boron compounds have a wide spectrum of antimicrobial features against bacteria, yeast, and fungus species. Especially, boric acid and sodium borate were shown to be effective against *S. aureus, K. pneumoniae, C. albicans,* and *A. niger* microorganisms [43,44,45,46]. Previous studies related to boron-based wound healing approaches mainly focused on boric acid and borax applications [8,40,47,48]. This project is the first study that investigates wound healing and antimicrobial properties of modified hBN and B_4_C nanoparticles in a comprehensive manner.

## 5. Conclusions

There is an immediate need to discover alternative treatments for chronic wound healing. Advanced applications like growth factor treatments or engineered tissue transplantation require costly equipment, time, and special facilities to apply these treatments. Thus, the discovery of novel compounds that are capable of regeneration to directly treat wounds is crucial. Here, we investigated the wound-healing potential of hBN-ALA and B_4_C-ALA nanoparticles. We showed that both compounds have good regenerative properties on the HDFa cell culture, promising antimicrobial features against *E. coli* and *S. aureus* bacterial strains, and favorable antioxidant properties that may contribute to the wound-healing process. Finally, we observed that ALA molecule integration enhances significantly both regenerative and antimicrobial properties of hBN and B_4_C nanoparticles. In conclusion, our results indicated that hBN-ALA and B_4_C-ALA conjugates are promising candidates as wound-healing and antimicrobial agents in chronic wound treatment. Also, animal wound healing models should be used to understand comprehensive wound-healing properties of hBN-ALA and B_4_C-ALA nanoparticles. On the other hand, in vitro scratch assay is a fast and reliable test to analyze the regenerative properties of nanoparticles in an effective manner.

## Figures and Tables

**Table 1:** Effects of hBN, hBN-ALA, B_4_C, B_4_C-ALA, and ALA on TAC and TOS levels in HDFa cell culture at 24 h. Symbol (*) represents statistically significant difference (*p* ≤ 0.05) compared to the negative control.

Groups	TAC (mmol Trolox Equiv./L)	TOS (µmol H_2_O_2_ Equiv./L)
Control	0.081 ± 0.003	1.052 ± 0.002
hBN (50 µg/mL)	0.211 ± 0.006 *	1.052 ± 0.004
hBN-ALA (50 µg/mL)	0.261 ± 0.007 *	1.063 ± 0.008
B_4_C (50 µg/mL)	0.158 ± 0.031 *	1.032 ± 0.001
B_4_C-ALA (50 µg/mL)	0.161 ± 0.031 *	0.990 ± 0.006
ALA (50 µg/mL)	0.224 ± 0.055 *	1.019 ± 0.001

## Data Availability

The data presented in this study are available on request from the corresponding author. The data are not publicly available due to privacy.

## References

[1] Baktir G. (2020). Wound Repair and Experimental Wound Models. Experimed.

[2] Robson M.C., Steed D.L., Franz M.G. (2001). Wound healing: Biologic features and approaches to maximize healing trajectories. Curr. Probl. Surg..

[3] Broughton G., Janis J.E., Attinger C.E. (2006). The basic science of wound healing. Plast. Reconstr. Surg..

[4] Guo S., DiPietro L.A. (2010). Critical review in oral biology & medicine: Factors affecting wound healing. J. Dent. Res..

[5] Sandhiya S., Dkhar S.A., Surendiran A. (2009). Emerging trends of nanomedicine—An overview. Fundam. Clin. Pharmacol..

[6] Losi P., Briganti E., Magera A., Spiller D., Ristori C., Battolla B., Balderi M., Kull S., Balbarini A., Di Stefano R. (2010). Tissue response to poly(ether)urethane-polydimethylsiloxane-fibrin composite scaffolds for controlled delivery of pro-angiogenic growth factors. Biomaterials.

[7] Tong C., Zhong X., Yang Y., Liu X., Zhong G., Xiao C., Liu B., Wang W., Yang X. (2020). PB@PDA@Ag nanosystem for synergistically eradicating MRSA and accelerating diabetic wound healing assisted with laser irradiation. Biomaterials.

[8] Nzietchueng R.M., Dousset B., Franck P., Benderdour M., Nabet P., Hess K. (2002). Mechanisms implicated in the effects of boron on wound healing. J. Trace Elem. Med. Biol..

[9] KONCA M., KORKMAZ M. (2020). Comparison of Effects of Administration of Oral or Topical Boron on Wound Healing and Oxidative Stress in Rats. Kocatepe Vet. J..

[10] Blech M.F., Martin C., Borrelly J., Hartemann P. (1990). Traitement des plaies profondes avec perte de substance. interet d’une solution d’acide borique A 3 P. 100. Press. Med..

[11] Litovitz T.L., Klein-Schwartz W., Oderda G.M., Schmitz B.F. (1988). Clinical manifestations of toxicity in a series of 784 boric acid ingestions. Am. J. Emerg. Med..

[12] Linden C.H., Hall A.H., Kulig K.W., Rumack B.H. (1986). Acute ingestions of boric acid. J. Toxicol. Clin. Toxicol..

[13] Brufani M., Figliola R. (2014). (R)-α-lipoic acid oral liquid formulation: Pharmacokinetic parameters and therapeutic efficacy. Acta Biomed..

[14] Yıldırım Ö.Ç., Arslan M.E., Öner S., Cacciatore I., Di Stefano A., Mardinoglu A., Turkez H. (2022). Boron Nitride Nanoparticles Loaded with a Boron-Based Hybrid as a Promising Drug Carrier System for Alzheimer’s Disease Treatment. Int. J. Mol. Sci..

[15] Aydin N., Turkez H., Tozlu O.O., Arslan M.E., Yavuz M., Sonmez E., Ozpolat O.F., Cacciatore I., Di Stefano A., Mardinoglu A. (2022). Ameliorative Effects by Hexagonal Boron Nitride Nanoparticles against Beta Amyloid Induced Neurotoxicity. Nanomaterials.

[16] Türkez H., Arslan M.E., Mardinoğlu A. (2019). Pivotal role of micronucleus test in drug discovery. Micronucleus Assay: An Overview.

[17] Wang J., Xia Q. (2014). Alpha-lipoic acid-loaded nanostructured lipid carrier: Sustained release and biocompatibility to HaCaT cells in vitro. Drug Deliv..

[18] Wang J., Wang H., Zhou X., Tang Z., Liu G., Liu G., Xia Q. (2012). Physicochemical characterization, photo-stability and cytotoxicity of coenzyme Q10-loading nanostructured lipid carrier. J. Nanosci. Nanotechnol..

[19] Wetzler C., Kämpfer H., Stallmeyer B., Pfeilschifter J., Frank S. (2000). Large and Sustained Induction of Chemokines during Impaired Wound Healing in the Genetically Diabetic Mouse: Prolonged Persistence of Neutrophils and Macrophages during the Late Phase of Repair. J. Investig. Dermatol..

[20] Braun L.R., Fisk W.A., Lev-Tov H., Kirsner R.S., Isseroff R.R. (2014). Diabetic Foot Ulcer: An Evidence-Based Treatment Update. Am. J. Clin. Dermatol..

[21] Kar Y., Şen N., Demirbaş A. (2006). Boron minerals in turkey, their application areas and importance for the country’s economy. Miner. Energy-Raw Mater. Rep..

[22] Emanet M., Sen Ö., Taşkin I.Ç., Çulha M. (2019). Synthesis, Functionalization, and Bioapplications of Two-Dimensional Boron Nitride Nanomaterials. Front. Bioeng. Biotechnol..

[23] Khaliq H., Juming Z., Ke-Mei P. (2018). The Physiological Role of Boron on Health. Biol. Trace Elem. Res..

[24] Demirci S., Doğan A., Aydın S., Dülger E.Ç., Şahin F. (2016). Boron promotes streptozotocin-induced diabetic wound healing: Roles in cell proliferation and migration, growth factor expression, and inflammation. Mol. Cell. Biochem..

[25] Türkez H., Arslan M.E., Sönmez E., Geyikoğlu F., Açıkyıldız M., Tatar A. (2019). Microarray assisted toxicological investigations of boron carbide nanoparticles on human primary alveolar epithelial cells. Chem. Biol. Interact..

[26] Türkez H., Arslan M.E., Sönmez E., Açikyildiz M., Tatar A., Geyikoğlu F. (2019). Synthesis, characterization and cytotoxicity of boron nitride nanoparticles: Emphasis on toxicogenomics. Cytotechnology.

[27] Küçükdoğru R., Türkez H., Arslan M.E., Tozlu Ö.Ö., Sönmez E., Mardinoğlu A., Cacciatore I., Di Stefano A. (2020). Neuroprotective effects of boron nitride nanoparticles in the experimental Parkinson’s disease model against MPP+ induced apoptosis. Metab. Brain Dis..

[28] Stodolak-Zych E., Gubernat A., Ścisłowska-Czarnecka A., Chadzińska M., Zych Ł., Zientara D., Nocuń M., Jeleń P., Bućko M.M. (2022). The influence of surface chemical composition of particles of boron carbide powders on their biological properties. Appl. Surf. Sci..

[29] Singh P., Kaur M., Singh K., Meena R., Kumar M., Yun J.-H., Thakur A., Nakagawa F., Suzuki M., Nakamura H. (2021). Fluorescent boron carbide quantum dots synthesized with a low-temperature solvothermal approach for boron neutron capture therapy. Phys. E Low-Dimens. Syst. Nanostruct..

[30] Xu Z., Han S., Gu Z., Wu J. (2020). Advances and Impact of Antioxidant Hydrogel in Chronic Wound Healing. Adv. Healthc. Mater..

[31] Li Z., Zhang J., Fu Y., Yang L., Zhu F., Liu X., Gu Z., Li Y. (2020). Antioxidant shape amphiphiles for accelerated wound healing. J. Mater. Chem. B.

[32] Fitzmaurice S.D., Sivamani R.K., Isseroff R.R. (2011). Antioxidant Therapies for Wound Healing: A Clinical Guide to Currently Commercially Available Products. Skin Pharmacol. Physiol..

[33] Sinha M., Mardinoglu A., Ghose J., Singh K. (2020). Editorial: Redox Homeostasis and Cancer. Oxid. Med. Cell. Longev..

[34] Rymon-Lipinski T., Fichtner R., Benecke T. (1992). Study of the oxidation protection of MgO-C refractories by means of boron carbide. Steel Res..

[35] Rymon-Lipinski T., Wolf P. (1993). Reaction processes in the interior of an MgO-carbon brick with boron carbide additive. Steel Res..

[36] Li Y., Yang M., Xu B., Sun Q., Zhang W., Zhang Y., Meng F. (2018). Synthesis, structure and antioxidant performance of boron nitride (hexagonal) layers coating on carbon nanotubes (multi-walled). Appl. Surf. Sci..

[37] Xu Z., Chen Y., Li W., Li J., Yu H., Liu L., Wu G., Yang T., Luo L. (2018). Preparation of boron nitride nanosheet-coated carbon fibres and their enhanced antioxidant and microwave-absorbing properties. RSC Adv..

[38] Benderdour M., Van Bui T., Hess K., Dicko A., Belleville F., Dousset B. (2000). Effects of boron derivatives on extracellular matrix formation. J. Trace Elem. Med. Biol..

[39] Benderdour M., Hess K., Dzondo-Gadet M., Nabet P., Belleville F., Dousset B. (1998). Boron Modulates Extracellular Matrix and TNFα Synthesis in Human Fibroblasts. Biochem. Biophys. Res. Commun..

[40] Tepedelen B.E., Soya E., Korkmaz M. (2016). Boric Acid Reduces the Formation of DNA Double Strand Breaks and Accelerates Wound Healing Process. Biol. Trace Elem. Res..

[41] Gölge U.H., Kaymaz B., Arpaci R., Kömürcü E., Göksel F., Güven M., Güzel Y., Cevizci S. (2015). Effects of Boric Acid on Fracture Healing: An Experimental Study. Biol. Trace Elem. Res..

[42] Chebassier N., Ouijja E.H., Viegas I., Dreno B. (2004). Stimulatory Effect of Boron and Manganese Salts on Keratinocyte Migration. Acta Derm. Venereol..

[43] Li S., Wu C., Lv X., Tang X., Zhao X., Yan H., Jiang H., Wang X. (2012). Discovery of ferrocene-carborane derivatives as novel chemical antimicrobial agents against multidrug-resistant bacteria. Sci. China Chem..

[44] Yılmaz M.T. (2012). Minimum inhibitory and minimum bactericidal concentrations of boron compounds against several bacterial strains. Turk. J. Med. Sci..

[45] Totani T., Aono K., Yamamoto K., Tawara K. (1981). Synthesis and in vitro antimicrobial property of o-carborane derivatives. J. Med. Chem..

[46] Fink K., Uchman M. (2021). Boron cluster compounds as new chemical leads for antimicrobial therapy. Coord. Chem. Rev..

[47] Cencetti C., Bellini D., Pavesio A., Senigaglia D., Passariello C., Virga A., Matricardi P. (2012). Preparation and characterization of antimicrobial wound dressings based on silver, gellan, PVA and borax. Carbohydr. Polym..

[48] Rezvan G., Pircheraghi G., Bagheri R. (2018). Curcumin incorporated PVA-borax dual delivery hydrogels as potential wound dressing materials—Correlation between viscoelastic properties and curcumin release rate. J. Appl. Polym. Sci..

